# Adverse Events Profile of PrePex a Non-Surgical Device for Adult Male Circumcision in a Ugandan Urban Setting

**DOI:** 10.1371/journal.pone.0086631

**Published:** 2014-01-28

**Authors:** Moses Galukande, Kevin Duffy, Jean Paul Bitega, Sam Rackara, Denis Sekavuga Bbaale, Florence Nakaggwa, Teddy Nagaddya, Nick Wooding, Monica Dea, Alex Coutinho

**Affiliations:** 1 International Hospital Kampala, Kampala, Uganda; 2 International Medical Group, Kampala, Uganda; 3 Kanombe Hospital, Kigali, Rwanda; 4 Surgery Department, Mulago Hospital, Kampala, Uganda; 5 Infectious Diseases Institute, Makerere University, Kampala, Uganda; 6 International Health Sciences University, Kampala, Uganda; 7 Centre of Disease Control, Kampala, Uganda; Johns Hopkins University Bloomberg School of Public Health, United States of America

## Abstract

**Background:**

Safe Male Circumcision is a proven approach for partial HIV prevention. Several sub Saharan African countries have plans to reach a prevalence of 80% of their adult males circumcised by 2015. These targets require out of ordinary organization, demand creation, timely execution and perhaps the use of SMC devices.

**Objective:**

To profile Adverse Events rate and acceptance of PrePex, a non surgical device for adult male circumcision.

**Methods:**

A prospective study, conducted at International Hospital Kampala, Uganda, between August and October 2012. Ethical approval was obtained from Uganda National Council of Science and Technology.

**Results:**

Of 1,040 men received to undergo SMC, 678 opted for PrePex, 36 were excluded at an initial physical examination screening. 642 were enrolled and consented, and another 17 were excluded before device placement. 625 underwent the procedure. Average age was 24 years (±7). Twelve moderate AEs occurred among 10 participants 12/625, (1.9%). These were all reversible. Five had device displacement, one had an everted foreskin; five had bleeding after the device was removed and one had voiding difficulties. The majority (279 out of 300) of men interviewed complained of some pain within the week of placement. Mean pain score at device placement (using visual analogue scale) was 0.5, at device removal 4.5 and within 2 min of removal the pain score was 1.4. Over 70% of the devices were placed and removed by non-physician clinicians. Presented with a choice, 60% of men chose PrePex over surgical SMC. Close to 90% would recommend the device to their friends. Odour from the necrotic skin was a concern. Removals done 1–2 days earlier than day 7 were beneficial and conferred no extra risk.

**Conclusion:**

AEs of a moderate or severe nature associated with PrePex were low and reversible. PrePex is feasible for mass safe male circumcision scaling up.

## Introduction

In 2011, UNAIDS estimated that more than 34 million people living with HIV, 1.7 m died from an AIDS related disease and 2.5 million became newly infected with HIV. The HIV/AIDS epidemic is continuing to grow; the number of those infected is increasing by more than 500,000 per annum [1]. Discovering how to prevent the transmission of HIV is of primary concern to health care authorities worldwide [2].

It is well known from a range of observational and epidemiological studies that the lifetime risk of acquiring HIV among males can be significantly reduced by approximately 60% through safe male circumcision (SMC). Numerous papers on the topic have been published over the past two decades to elevate HIV prevention awareness, especially in sub-Saharan countries [3], [4], [5].

In 2009, the US Government (USAID) reported that scaling up male circumcision to reach 80 percent of adult males in 14 African countries by 2015 could potentially avert more than 4 million adult HIV infections between 2009 and 2025 and yield annual cost savings of US$1.4–1.8 billion after 2015, with a total net savings of US$20.2 billion between 2009 and 2025 [6], [7].

Today, there are over 38 million adolescent and adult males in East and southern Africa who could benefit from safe male circumcision for HIV prevention. The challenge Africa faces is how to safely scale up a surgical procedure in resource limited settings [6].

Uganda has a national plan to offer voluntary SMC to 4.2 million adult men in 5 years, as part of a comprehensive HIV prevention strategy. For the past 18 months several organizations, referred to as Implementation Partners (IPs), have performed approximately 389,000 SMCs, far fewer than the 820,000 required per annum to meet the 4.2 m target. Approaches beyond business as usual are urgently needed to realize scale up and reach the set targets.

PrePex is an elastic ring controlled radial compression device, designed to effect non-surgical male circumcision. It was developed to facilitate rapid scale up of non-surgical adult male circumcision in resource limited settings. It is easy to apply and offers some advantages the conventional surgical circumcision doesnt [8], [9], [10], [11].

The purpose of this study was to profile the adverse events when using PrePex.

## Methods

### Study design

One-arm, open label, prospective study to verify the safety of the non-surgical PrePex™ device for adult male circumcision with no injected anesthesia, performed by physicians (general surgeons and medical officers) and non physician clinicians (clinical officers and nurses).

### Study setting

This study was conducted from August to October 2012 at an urban SMC site at International Hospital Kampala (IHK), a private facility in Uganda's capital. The population of Kampala by day is estimated to be 2.5–million people [12].

### Study population

All males were scheduled to undergo voluntary circumcision in an effort to prevent the spread of HIV in resource limited high prevalence settings. No marketing or demand creation activities for PrePex were done. In the past 18 months the majority of clients presenting for SMC came from within 10km proximity of the IHK SMC site.

### Study duration

Study duration per subject was up to 8 weeks (including follow up). The follow up was conducted by telephone, face-to-face scheduled visits and unscheduled visits if necessary, as judged by the patient or the Principal Investigator.

### Inclusion criteria

Participants included were those: aged –18 to 49 years, eligible adult males wishing to be circumcised, agreed to abstain from sexual intercourse for 6 weeks after device removal, agreed to abstain from masturbation for 2 weeks after device removal, agreed to perform follow up via telephone (or physical review if applicable) and those who were able to comprehend and freely give informed written consent for participation in this study and were considered by the investigators to have good compliance for the study.

### Exclusion criteria

Subjects excluded were those: with active genital infection, (e.g. genital ulcers, urethral discharges), and those with penile abnormalities deemed unfit for device placement such as frenulum breve, hypospadias, phimosis, paraphimosis, warts under the prepuce and epispadias. Those known to have bleeding or coagulation abnormalities; other co-morbidities such as hypertension, uncontrolled diabetes, mental illnesses and those whose prepucial openings could not accommodate the inner plastic ring were also excluded.

### The device

The PrePex device consists of an inner plastic ring, an outer elastic ring, a placement guide and a verification thread; there is also a sizing template with 5 holes of differing sizes to guide the selection of the device size. The device works by compressing the foreskin so as to cut off circulation distally, after which the distal foreskin becomes necrotic allowing relatively easy removal. All components were created for single use and disposal [9].

### Procedure

Upon receiving all men desirous of getting circumcised, a group counselling session was conducted by the Principal Investigator and assistants. During the 30–45 minutes session, information was shared using visual aids, and questions were asked and answered. The analogy given for necrotic tissue was the traditional removal of an extra digit in infants using a string to tie it off. The shared information included but was not limited to: the rationale for SMC, the barriers to SMC for men in Uganda including fear of pain and cost of time off work, the need for innovation to scale up SMC, the process and how surgical SMC is conducted, how the PrePex device contrasts with the surgical method, demonstration with pictorial aids for the placement and removal process, the possible mild, moderate and severe AEs including pain and odour. After care instructions for genital hygiene, abstinence and the availability and use of the hotline provided. Participants were given the opportunity after this session to choose between PrePex and the surgical method. Those who chose PrePex were taken into a screening room to be assessed for eligibility. If eligible, and after the subject signed the informed written consent form (one-on-one with the principal investigator or designee), they were then enrolled into the study.

On an examination bed in street clothes, clients lowered their trousers/shorts down to the knees. The genitals were swabbed with 0.5% chlorohexidine. The penile girth was measured using a single use disposable card with holes representing 5 sizes (A–E), A being the smallest and E the largest, after which the appropriately sized device was placed. One of the precautions was to check that no foreskin was folded under in the inner ring, a known cause for post placement oedema and pain. Before discharge the clients were counseled about post placement care. Emphasis was placed on the need to continue protecting themselves and their partners against HIV after circumcision and on the importance of allowing the penis to fully heal before sexual activity is resumed (6 weeks after removal).

The measure of pain was gauged using the visual analogue pain scale (VAS), a range of 0–10. The numbers 0, 2, 4, 6, 8 and 10 are accompanied by face drawings that correspond to the numbers, the participant selects one that best represents how they feel. These scores were captured during four different stages of the study: during placement, post placement, during removal and post removal, at both scheduled and unscheduled visits.

Follow up included a phone call on day 1 after placement, a scheduled visit on day 5–7 for removal and on day 14, with a further phone call. The phone calls were made by trained nurses and counselors. A 24-hour hotline team was available for unscheduled visits and calls. The clients who were unscheduled were attended to by trained nurses, the principal investigator and or designated co principal investigator.

### Data collection

A pretested structured Case Report Form (CRF) was used to collect data. The questionnaires were filled in by research attendants and all designated and trained device operators. Data was collected at placement, removal and follow up for all 625 clients. In addition, 300 participants were interviewed after removal to gather information on odour, pain and acceptability of the device. The choice of the 300 interviews for exit interveiw was by convenient sampling and logistical considerations but more important was the need to collect more data on odour, a variable which was not pre-planned of the study but was incoprated (IRB amendemnet approval was obtained) after noticing that odour was a concern. A structured pretested questionnaire was used and administered by trained research assistants (that were social science graduates).

### Study variables and AE classification

#### Socio-demographics, adverse events (AEs)

The Global Harmonisation Taskforce (DHTF) [13] and the international organization for standardization define an AE as: ‘Any untoward medical occurrence, unintended disease or injury, or clinical signs (including an abnormal laboratory findings in subjects, users or other persons, whether or not related to the investigational medical device’. AE classification was by timing in relationship to device placement, wearing, removal and after removal. AEs were classified as mild, moderate or severe and included: pain as assessed with VAS); bleeding; analgesic/anaesthesia related; damage to penis (bruise, or abrasion); difficulty with placement but placement failure was not considered as an AE; infection (erthyema, purulent discharge, cellulitis/necrosis); haematoma; problems voiding device displacement; self removal; early removal; difficulty in removal; and wound dehiscence [14]. Mild AE were adverse events (AEs) that resolved without requiring any intervention, moderate AEs, were those events not classified as serious/severe AE but required an intervention by a health care provider or medication (parental, oral or tropical). Serious/severe AE are events that led to death or led to a serious deterioration in health of patients, or resulted in a life threatening illness or injury, resulted in a permanent injury of a body structure or function, required inpatient hospitalization or prolongation of existing hospitalization or resulted in medical or surgical intervention to prevent permanent impairment to body structure or body function [15].

### Ethical consideration

This study obtained approval from the Makerere School of Medicine Research and Ethics Committee and the Uganda National Council of Science and Technology. Written Informed consent was obtained from all participants. Available to all participants, was the required minimum HIV prevention package which included risk reduction counseling, STI treatment and condom distribution, this service available at the study site at all times and was provided by trained nurses and counsellors.

## Results

In all 625 adult males underwent the procedure and were included into the study. Their mean age was 24 years, the age range was 18–49 years, other demographic parameters included, Education status: those at Tertiary level were 34%, Secondary was 50% and Primary level were 16% as shown in table 1.

**Table 1:** Baseline characteristics of study participants, IHK Uganda PrePex trial study 2012.

Variable	Number (percentage)
Mean age	24 sd 7
Age range	18–49 years
**Education**	
Tertiary	212 (34%)
Secondary	312 (50%)
Others	101 (16%)
**HIV prevalence**	3 (0.5%)
**Occupation**	
Students	63 (10%)
*Boda boda cyclists	6 (1%)
Others	556 (89%)
**Penile sizes** *(24–36mm)*	
A	61 (10%)
B	171 (28%)
C	224 (35.5%)
D	113 (18%)
E	52 (8%)
Missing data	4 (0.5%)
**Screen failure**	
Screen failure	51/678 (8%)
Clients excluded at initial physical screen before consent	36
Narrow fore skin	27
Frenulunm breve	4
Client withdrawal	2?
Penile ulcer	1
Penile wart	1
Hypospadia	1
**Clients admitted to study but device not placed**	
Lesion on glans	1
Adhesions	1
Narrow foreskin	4
Repeated erections during procedure	1
< size A	1
Frenulum breve	1
**Withdrawals before placement**	
Below age	1
Withdrawals on request (changing their mind)	7?

* boda boda refers to motorcycles a common and popular two wheel means of transport for mostly short distances in the country ? Exclusions due to change of client mind not included in screen failure rates.

Mild AEs were mostly due to short lived pain during device removal and required no intervention, the pain lasted less than 2 minutes, 99/625 (15.8%) had pain scores of 8 or above on the visual analogue scale of 0 to 10 (VAS), see table 2.

**Table 2:** Adverse events profile IHK PrePex Uganda study 2012.

Timing	Adverse Event	Values	Comments
**Events during placement**	Pain *n = 625*	0.5 (average score – in VAS 0–10)	Short lived <2min (considered Mild AE).
	Bleeding *n = 625*	Nil	-
	Others	Nil	-
**Events during wearing**	Pain *n = 300*		Pain/discomfort was mostly tolerable. Scores of 10 were considered mild AE, clients were encouraged to carry on with analgesics previously given
	VAS Pain scores	n (%)	
	0	19 (6.3%)	
	2	219 (73%)	
	4	25 (8%)	
	6	21 (7%)	
	8	14 (5%)	
	10	2 (0.7%)	
	Odour *n = 300*		
	Odour complaints	238/300 (79%)	Not considered an AE but a side effect.
	Smell by day of wearing	Clients noticing smell	Odour for the majority (63%) was noticed on D3 and 4.
	Day 2	18 (8%)	
	Day 3	68 (28%)	
	Day 4	83 (35%)	
	Day 5	40 (17%)	
	Day 6	25 (10%)	
	Day 7	4 (2%)	
	Early removals *n = 625*		Eight D4 removals were done in error when D4 was mistaken by the client and operator for D5
	Day 4	12	
	Day 5	55	
	Day 6	86	
	Device displacement *n = 625* SAE	5 (1%)	Two had sex, one on D2 and the other D4. Displacements were mostly due to tampering with the device.
	Transient voiding difficulties *n = 300* (Mild-Moderate AEs)	34/300 (11%)	All were able to void without intervention except one who used a razor blade to open up the dry necrotic foreskin. All were considered mild AEs. Save one which was considered moderate.
	Device partial self detachment *n = 300*	66/300 (22%)	Partial detachment exposes raw surface that is thought to contribute to high pain scores during device removal. No additional analgesics were given during removal as pain was short lived (Mild AE)
	Pseudoparaphimosis* *n = 625*	1	A new event that required a surgeon's intervention (classified as moderate AE).
	Clients engaging in sexual intercourse *n = 300*	6/300 (2%)	These clients did not heed the counsel of abstinence
**Events during removal**	Pain *n = 625*		Considered mild AE
	Those with scores ≥8	99/625 (16%)	
	Over all pain score	4 (average score – in VAS 0–10)	Pain short lived ≤2 minutes
	Bleeding *n = 625*	4 required suture control and 1 required pressure control	Both Moderate AEs
**Events during entire period**	Unscheduled visits *n = 625*	15	These were for various reasons; pain, odour and convenience. For pain, clients were encouraged to take analgesics as previously prescribed. These clients did have the devices removed from private clinics because they couldn't return due to lack of time and the other had a car accident and reported that the device fell off, foreskin was removed in a private clinic
	Those that didn't return for device removal	*2*	

* This was deemed the appropriate term for retracted necrotic dry foreskin causing pain and covering the outer black device ring, therefore posing a challenge of removal.

There were 15 unscheduled visits 15/625 (2.4%). There was multiplicity of AEs for some clients, 12 clients had 2 AEs, 1 client had 3 AEs and I had 4 AEs. Five AEs were associated with premature device displacement; two of these, admitted attempting to have sexual intercourse while wearing the device on day 2 and day 4 respectively.

One purposefully removed the device one day after placement because he had a party that day and thought the device might get in the way of the party proceedings. The other was a 19 year old who related a story that his friend pulled the device off him.

For all the device displacement cases, a formal surgical SMC was performed uneventfully and the AEs were resolved.

Five clients bled immediately after removal of the device. The nature of the bleeding required a stitch or two to achieve haemostasis. Three of these had spurting vessels.

One of the clients admitted to prior treatment for a non specified coagulopathic disorder he had not disclosed at pre SMC counseling and screening.

Considering the 12 moderate/severe AEs (that occurred among 10 participants), the following were their baseline characteristics: the mean age was 30 years, the range was 18–42 years, educational level (5 had attained secondary level, and 5 had attained university level), and for occupations; 2 were students, 2 unemployed and 6 were in formal gainful employment.

We noted among the 300 exist interviews that for all who experienced pain/discomfort, it started on days 2–3. 90% reported control of pain by the analgesia given. 93% (279/300) used the pain killers given, 52% (156/300) took all the pain killers given and 13% (38/300) had routine activities disrupted due to pain. 16/300 (5%) reported pain scores of 8–10 while wearing the device.

Seventy nine percent (238/300) of the clients interviewed after removal reported bad odour. Exploring this further, only 3 out of the 300 participants interviewed indicated that another person had told them they ‘smelt bad’. No formal odour scale was used to gauge odour intensity.

The majority of men, 99% (623/625), returned to have the device removed within the allowable 5–7 days after replacement.

In total, 44 of 678 who had originally chosen PrePex were disqualified on clinical grounds making a screen failure rate of 6.5%.

The majority of participants at the exit interviews after device removal [268/300 (89%)] answered in the affirmative if they would recommend the device to a friend.

## Discussion

This study set out to profile the adverse events associated with the PrePex device, an elastic ring controlled radial compression device for non-surgical adult male circumcision. The PrePex device was developed to facilitate rapid scale up of non-surgical adult male circumcision in resource limited settings. We found the moderate to severe adverse events rate was less than 2%. Mild AEs were mostly due to short lived pain during device removal, the pain lasted less than 2 minutes. Although there had been attempts to standardize terminology and classification of adverse events in studies of conventional male circumcision and circumcision devices, the classification schemes are evolving as more information about the types and timing of AEs become available. The different mechanisms of actions of the devices and the differences from conventional surgical circumcision techniques have led to differences in the types of AEs and characterization of the AEs [13], [15].

Unscheduled visits prior to day 7 occurred and are to be expected with future use of the device. Odour was a problem that was noted by the men and occasionally by others around.

Device displacement in four out of the five cases was due to device manipulation, even though all participants were well informed about the need to avoid manipulating the device, manipulation included purposeful removal of the device or engaging in sex activities despite prior counseling. Device displacement required surgical intervention to pre-empt further complication, on this basis a classification of severe AE was made.

Out of the 300 exit interviews conducted immediately after the device removal, six participants admitted to attempting penetrative vaginal sex during the week of wearing the device. The number 6 out of 300 (2%) may be an underestimate as men may have been reluctant to disclose this information. But also we did not follow up the sex resumption issue beyond 14 days. Studies in Zambia and Kenya indicated a significant percentage (24–31%) of circumcised men resuming sexual intercourse before the mandatory 6 weeks abstinence period recommended to allow full healing of the penis [16], [17].

This early resumption of sex prior to healing raises the question, there could be an increased risk of HIV acquisition through a wound that is not completely healed, infections acquired during a short period of potential increased vulnerability are far outweighed by the number of HIV infections averted over subsequent years [16], [17]. Fully understanding the factors that lead to early resumption of sex after circumcision would inform preventive interventions. Learning from the men that adhere to abstinence may be valuable. We paid attention to the right messaging, emphasizing no sex before 6 weeks, not even with a condom. We emphasized the fact that some or many will indeed look healed, with no pain and no open wound long before six weeks elapse but that does not imply that it is safe to resume sexual intercourse; for PrePex the instructed period of abstinence was 6 weeks after device removal.

For all the device displacement cases, a formal surgical SMC was performed uneventfully and the AEs were resolved. This experience suggests that, in the context of program implementation, there should be a service available to manage AEs. Either a PrePex only center with a functional referral pathway to a center that is capable of performing a surgical SMC or all PrePex service sites should have the capability to offer both services 24/7.

### Bleeding

Five clients bled immediately after removal of the device. The nature of the bleeding among four required a stitch or two to achieve haemostasis. Three of these had spurting vessels, likely to be arterial, from the under lying granulation tissue and perhaps this was due to disruption of granulation tissue caused by either the spatula ‘digging’ during the process of device removal or in the process of excising the necrotic foreskin when the granulation tissue/normal skin edge is disrupted by the sometimes inadvertent pull and tag action.

The personnel managing these events were capable of applying haemostatic stitches. The programmatic implications of this are that the AE managing personnel should be capable of performing suture haemostasis.

One of the clients admitted to prior treatment for a non specified coagulopathic disorder he had not disclosed at pre SMC counseling and screening. Attention should be paid to screen out individuals that may have bleeding disorders.

### Counting adverse events

Adverse events were determined according to the AE definition and classification, which defines an adverse event as an unexpected occurrence that either endangers the life of the client or disrupts normal life activities or causes an unintended consumption of resources, staff or materials [18], [19].

Counting AEs was approached in two ways, first, counting of individuals among whom any AE(s) occurs i.e. clients with AEs. Secondly, the number of AEs occurring at one point or over time in the same individual for the entire study period. For example one of the clients in the study returned 2 days earlier due to pain (an unscheduled visit) this was (AE = 1). For the same individual, the device was removed earlier than planned (AE = 2), during the removal he had a pain score of 8, (AE = 3 but mild) and after removal he bled and required a stitch for haemostasis (AE = 4). How these should be reported is subject to debate. Whereas this approach could be viewed as inflating the AE rate it gives a clear sense of what could happen and thus perhaps better informs program planning. We also described an event that had not been reported before, the necrotic foreskin retracting everting and in the process obscuring the outer ring, posing a challenge during removal; we termed this pseudo-paraphimosis.

There were some other events which are not yet classified as AEs, but are worth noting. One client, six weeks after removal, reported that his penis appeared bigger than before. He now requires a king sized condom yet previously he wore normal sized ones. Another client reported noticing a bend during erection which he associated with some coital difficulties though these were eased by the application of a lubricant pre-coitus. A physical examination revealed a normal scar and we adopted a wait and see approach. Larger sample size studies and longer surveillance periods may be required to exhaust all possibilities of adverse events and side effects.

### Pain

At the beginning of the study, the group counseling messages did not dwell on the occurrence of pain or odour the expectations of the clients therefore were that no pain would occur. Our findings were that over 70% reported some pain or discomfort while wearing the device and of those who reported pain 10% rated it as moderate to severe (VAS scores of 8 and above).

As a result we changed the messaging to include some pain anticipation and informed clients that the use of PrePex may not be a completely pain free experience for all of them, that the experience would vary from one to another. All clients were provided with a 3 days' supply of ibruprofen 400 mg tablets and paracetamol 1 g tablets each taken thrice a day. We noted that for all who experienced pain/discomfort, it started on days 2–3. The majority reported control of pain by the analgesia given. A few had routine activities disrupted due to pain.

Placement was generally pain free. Removal procedures registered more pain. We observed that there was more pain among those who had partial self detachment of the device.

Most post placement pain started on day 2 and peaked on day 4. The pain may be related to inflammation due to skin necrosis. Early necrosis pain could have been reduced or masked by the use of lignocaine 5% topical applications. Overall the pain was tolerable and mostly classified as mild.

### Odour

Bad odout was not anticipated during the preparations for this study. Bad odour may be described as a discomfort occurring from use of the device and whether this is considered a mild AE or a side effect is subject to debate Bad odour could potentially make an otherwise excellent innovation less favourable. 63% (189/300) of the clients interviewed after removal reported bad odour. Exploring this further, only 3 out of the 300 participants interviewed indicated that another person had told them they ‘smelt bad’. When asked whether they would recommend the device to a friend 89.3% (268/300) responded in the affirmative suggesting that the odour and pain may not have had such a negative impact but, nonetheless, it remains important to seek means of mitigating both.

The odour may have been caused by anaerobes harboring in the wet necrotic foreskin. Further research and study will be needed to verify this conjecture. In some cases device removal was performed 1 to 2 days earlier than originally scheduled due to complaints about bad odour. Removal at day 5 may be one possible solution to help mitigate client concerns about odour. Another plausible solution is to emphasize good hygiene, washing with soap and dabbing dry, at least twice a day. Use of metronidazole gel or powder is another option to be investigated.

### Voiding difficulties

Voiding difficulties were encountered but these were mostly of transient nature. The drying or necrotizing prepuce gets in the ‘way’ and this ought to be considered in the pre-device placement counseling sessions to avoid clients taking it upon themselves to relieve real or perceived ‘obstructured’ as one did in this study using a razor blade.

### Choice (Acceptance of the device)

All clients were given an opportunity to choose between PrePex or the surgical method. Before choosing, they participated in a group counseling session during which the PrePex processes and outcomes were outlined using visual aids. Some of the highlights of this counseling session included: no injectable anaesthesia, no cutting of live skin, no bleeding, and an immediate return to work but with one extra week of abstinence compared to surgical SMC.

We established that in this device naïve community the immediate uptake of PrePex was 60% in favor before use. After device use 90% would recommend the device to their friends.

The reasons for this choice, or the lack of it, were varied. Some cited fear of being the first, others wanted to have the circumcision completed that day with no need to return for device removal and others preferred the tried and time tested surgical circumcision option. Some expressed a sense of feeling ‘ambushed’ with the information about the new device method.

There was a growing acceptance of the device by men in Kampala during the study period. The majority of men, 99%, returned to have the device removed within the allowable 5–7 days after replacement.

### Screen failure

In total, 44 of 678 who had originally chosen PrePex were disqualified on clinical grounds making a screen failure rate of 6.5%. The implication of this is in program planning as a proportion of individuals would not be suitable for the device even if they originally chose to have it.

### Study limitations

This study was conducted in an urban setting targeting young men aged between 18–49 years. It was conducted at a fixed site within a hospital setting with a surgically competent back up, team to handle AEs and screen failures and we need to extend this trial to rural settings and use the mobile operations model (SMC camps) in order to more fully explore acceptance and feasibility that would inform policy guidelines for such a device in Uganda's national SMC program.

Acceptance and choice between surgical male circumcision and PrePex may differ in rural settings. Attitudes and practices towards bad odour, hygiene and pain tolerance may be different too.

Whereas we assumed that the study population was device naive, some men could have come because their friends recommended the device therefore causing an over estimation of PrePex preference.

It was not possible to draw meangful inferences from the baseline characteristics of the men who had the moderate AEs as the number was small.

## Conclusion

PrePex as a non-surgical male circumcision device is a viable option for upscaling up of safe male circumcision with a low rate of AE and high acceptance rate by the clients (90% recommend its use). Use of Prepex would not exclude the conventional surgical circumcision since some may be screened out or may require surgical circumcision to address AEs. Further studies may be necessary to determine performance in rural and mobile contexts.
